# Lung cancer screening in occupational settings: a scoping review

**DOI:** 10.3389/fpubh.2026.1888829

**Published:** 2026-08-27

**Authors:** Edelqueen Ijeoma Okoro, Helena Keller, Felix Greiner, Jan Heidrich, Volker Harth

**Affiliations:** Institute for Occupational and Maritime Medicine (ZfAM), University Medical Center Hamburg-Eppendorf (UKE), Hamburg, Germany

**Keywords:** early cancer detection, low-dose computed tomography (LDCT), lung cancer screening, occupational health, workplace health surveillance, workplace screening programs

## Abstract

**Background:**

Lung cancer is the leading cause of cancer-related mortality worldwide. It is often asymptomatic until advanced stages, resulting in delayed diagnosis and poor survival. Screening enables early detection and reduces mortality. Multiple occupational and environmental exposures have been linked to lung cancer, and some guidelines recommend their inclusion in risk assessment. This review aims to map the existing evidence on lung cancer screening (LCS) in occupational settings.

**Methods:**

This review followed JBI methodology and employed a three-step search strategy across six databases: PubMed, Web of Science, Scopus, CINAHL (via EBSCO), ScienceDirect and Cochrane Library. Eligible studies were those reporting on LCS conducted in any occupational setting, published in English or German between 2013 and November 2024. Two reviewers independently screened studies for inclusion. Data were synthesized thematically, and findings were reported according to PRISMA-ScR guidelines.

**Results:**

Twenty-one studies from 12 countries were included, conducted across diverse occupational populations. Low-dose computed tomography (LDCT) was used for screening in 18 studies (15 as a standalone modality, 3 combined with biomarkers, sputum cytology, or chest ultrasonography respectively), while 3 studies employed alternative approaches. Eligibility criteria varied substantially, and intervention durations ranged from 3 months to 13 years. Individual and combined recruitment strategies were identified, including emails, invitation letters, face-to-face interactions, direct outreach by department heads or supervisors, and referrals from primary care or specialty clinics. Thematic synthesis yielded five overarching themes: (1) occupational exposures as lung cancer risk factors and limitations of current LCS guidelines; (2) lung cancer burden across occupational groups; (3) benefits and performance of LDCT in occupational cohorts; (4) tools and multimodal strategies to enhance LDCT; and (5) implementation, participation, and program feasibility.

**Conclusion:**

Occupational settings present significant opportunities for early lung cancer detection among high-risk workers, while offering potential benefits for those with low or uncharacterized exposures. However, the evidence base remains limited, with significant gaps in implementation protocols and reporting standards. Addressing these deficiencies requires robust prospective studies and standardized frameworks to ensure the translation of occupational LCS into a reproducible, scalable public health intervention.

**Systematic review registration:**

https://doi.org/10.17605/OSF.IO/UHS8D

## Introduction

Lung cancer is recognized as the leading cause of cancer-related deaths worldwide, accounting for approximately 1,817,469 deaths in 2022 (22.7% in men and 13.5% in women) (1). Often, the disease remains asymptomatic until advanced stages, resulting in late diagnosis and low survival rate. However, this major public health issue can be mitigated through screening, which involves testing asymptomatic individuals to identify lung cancer at an early stage. This early detection, combined with timely treatment, can significantly reduce mortality and improve patient health outcomes. Several randomized controlled trials (RCTs) and meta-analyses have demonstrated the efficacy of low-dose computed tomography (LDCT) in the early detection of lung cancer and subsequent mortality reduction, most prominently, the US National Lung Screening Trial (NLST) and the Dutch-Belgian NELSON study (2, 3). LDCT has become the recommended method for lung cancer screening (LCS), against chest x-ray and various biomarkers previously used (4).

The NLST and NELSON defined high-risk persons solely by age and smoking history, hence informing the first LCS guideline of the US Preventive Services Task Force (USPSTF) in 2013, and the subsequent update in 2021 (5). However, it has been argued that the use of age and smoking as the sole risk assessment factors is limiting (6). Occupational carcinogens, including asbestos, silica, diesel exhaust, and radon, often act synergistically with tobacco smoke, substantially increasing lung cancer risk beyond the effect of either factor alone (7). Historical measures, such as the ban on asbestos in many countries, have demonstrated significant long-term health benefits (8, 9). Whilst primary prevention remains essential in reducing lung cancer burden, LCS could be beneficial especially in occupational settings where hazardous exposures persist.

Therefore, the US National Comprehensive Cancer Network (NCCN), the American Association for Thoracic Surgery, the 2015 Helsinki Consensus Report and a recent Collegium Ramazzini statement on occupational LCS have recommended the consideration of occupational exposures as potential risk factors for lung cancer (6, 10–13). Similarly, some risk prediction models have been developed that included asbestos exposure as a risk factor e.g. the Liverpool Lung Project model (LLP_v2_) and the Bach's model (14, 15).

Given the established link between various occupational and environmental exposures and lung cancer, occupational settings may represent an important setting for lung cancer early detection efforts. This would improve outreach to populations at high risk of lung cancer and boost participation in LCS by providing access to people of diverse socio-demographics, especially to employees who seldom seek regular medical care. The European Union (EU) has co-funded a project entitled “Strengthening the screening of lung cancer in Europe” (SOLACE), which aims to improve organized LCS in EU Member States by facilitating a structured implementation of LDCT LCS according to the individual needs of EU Member States (16). One of the key tasks of the SOLACE project is to implement LCS pilot projects across various populations, including those from occupational settings.

Despite the recognition of occupational settings as valuable platforms for LCS, the existing body of literature on this topic is limited, and focuses mainly on populations with a history of occupational asbestos exposure (6, 17–20). Comprehensive studies evaluating general workplace-based LCS programs are rare. Therefore, this scoping review aims to comprehensively map the available evidence on LCS in general occupational settings, examining screening implementation across different occupational populations, and identifying key concepts, outcomes, challenges, and recommendations from the studies.

## Methods

This scoping review was conducted in accordance with the Joanna Briggs Institute (JBI) methodology for scoping reviews (21), with adaptation to the Preferred Reporting Items for Systematic Reviews and Meta-Analyses extension for Scoping Reviews (PRISMA-ScR) guideline (22). An *a priori* protocol for the study, developed using the JBI (21) and the Lely et al. (23) scoping review protocol guidelines, was registered and published in Open Science Framework (24). Eligibility Criteria is shown in Table 1.

**Table 1:** Eligibility criteria.

Domain	Inclusion criteria	Exclusion criteria
Participants	Studies must involve LCS of workers	Studies of workers whose employment played no direct or indirect role in their participation in the LCS e.g. studies that retrospectively analyse occupation of LCS participants
Concept	Studies must clearly state the screening modalities e.g. use of either LDCT, biomarkers, other imaging technologies other than LDCT, or their combinations	
Context	Studies must have been conducted in any type of occupational setting in any country in the world	Studies of population–based LCS without clear recruitment from occupational settings
Types of evidence sources and design	Both primary and secondary sources, including randomized controlled trials, non–randomized controlled trials, observational studies, pilot studies, systematic reviews, etc. In addition, gray literature, particularly conference abstracts/proceedings were also considered.	Retrospective cohort studies that do not describe or evaluate the actual screening intervention (e.g., studies assessing occupational exposures or mortality)
Language	Only studies published in English or German language	Studies with English abstract but has full text in another language other than English or German
Year of publication	Only studies published between 2013 and 2024 (since USPSTF issued its first recommendation for LCS with LDCT in 2013)	

### Information sources and search strategy

A three-step search strategy was utilized for this review. First, a preliminary limited search of PubMed was conducted to identify relevant text words and index terms, which were used to develop an advanced search strategy. Second, this advanced search strategy was adapted for the other included databases: Web of Science, Scopus, CINAHL (via EBSCO), ScienceDirect and Cochrane Library. These database searches were conducted between 28/11/2024 and 07/12/2024, with the detailed search strings shown in Table 2. In addition to the electronic databases, a targeted Google search of websites and gray literature was conducted on 09/12/2024 utilizing the advanced search strings: ((Lung Cancer OR Lung Neoplasms) AND (Screening OR Early Detection of Cancer) AND (Workers OR Employees OR Occupational Groups OR Workplace OR “Occupational Setting)),” and screening the first 50 results for relevance. In line with the eligibility criteria, all searches were limited to studies published in English or German between 2013 and 2024 (year 2013 was selected as the baseline because it marks USPSTF's first recommendation for LCS using LDCT). Third, the snowballing method was applied by screening the reference lists of all included publications for additional relevant studies. EndNote 20 (Clarivate Analytics, PA, USA) was used for citation management. The Rayyan software program (Rayyan Systems Inc., Cambridge, MA, USA) was used for screening and study selection (25).

**Table 2:** Database search strategy.

Number	Search term
a. PubMed	
1	(Lung Cancer) OR (Lung Neoplasms[MeSH Terms])
2	(Screening) OR (Early Detection of Cancer[MeSH Terms])
3	((((Workers) OR (Employees)) OR (Occupational Groups[MeSH Terms])) OR (Workplace[MeSH Terms])) OR (“Occupational Setting”)
4	((#1) AND (#2)) AND (#3)
5	((#1) AND (#2)) AND (#3) Filters: English, German, from 2013–2024
b. Web of science	
1	(ALL=(Lung Cancer)) OR (ALL=(Lung Neoplasms)
2	(ALL=(Screening)) OR (ALL=(Early Detection of Cancer)
3	((((ALL=(Workers)) OR (ALL=(Employees)) OR (ALL=(Occupational Groups)) OR (ALL=(Workplace)) OR (ALL=(“Occupational Setting”)
4	#1 AND #2 AND #3
5	#1 AND #2 AND #3 Filters: English, German, from 2013–2024
c. Scopus	
1	Lung Cancer OR Lung Neoplasms
2	Screening OR Early Detection of Cancer
3	Workers OR Employees OR Occupational Groups OR Workplace OR “Occupational Setting”
4	#1 AND #2 AND #3
5	#1 AND #2 AND #3 Filters: English, German, from 2013–2024
d. CINAHL (Via EBSCO)	
1	(Lung Cancer) OR (MH “Lung Neoplasms”)
2	(MH “Cancer Screening”) OR (Early Detection of Cancer)
3	(Workers OR Employees OR Occupational Groups OR Workplace OR “Occupational Setting”)
4	((#1) AND (#2)) AND (#3)
5	((#1) AND (#2)) AND (#3) Filters: English, German, from 2013–2024
	^*^MH = Exact Subject Heading
e. Science direct	
1	(Lung Cancer OR Lung Neoplasms) AND (Screening OR Early Detection of Cancer) AND (Workers OR Employees OR Occupational Groups OR Workplace OR “Occupational Setting”) Filters: English, German, from 2013–2024
	^*^Selected Journals: European Journal of Cancer; The Lancet Oncology; Lung Cancer; CHEST; Journal of Thoracic Oncology; Annals of Oncology; Cancer Epidemiology; Journal of Geriatric Oncology; Clinical Lung Cancer
f. Cochrane library	
1	Lung Cancer
2	MeSH descriptor: [Lung Neoplasms] explode all trees
3	Screening
4	MeSH descriptor: [Early Detection of Cancer] explode all trees
5	Workers
6	Employees
7	MeSH descriptor: [Occupational Groups] explode all trees
8	MeSH descriptor: [Workplace] explode all trees
9	“Occupational Setting”
10	#1 OR #2
11	#3 OR #4
12	#5 OR #6 OR #7 OR #8 OR #9
13	#10 AND #11 AND #12
14	#10 AND #11 AND #12 Filters: English, German, from 2013–2024

All identified citations were collated and duplicates removed. For the pilot testing, a live calibration exercise was conducted by the two reviewers involved in both title/abstract and full-text screening. Using a random sample of 20 titles and abstracts, the team met synchronously via video conferencing to review and discuss each record collectively against the draft inclusion and exclusion criteria. Discrepancies in interpretation were debated in real-time until a 100% mutual consensus was reached for every record in the sample. This collaborative process allowed the team to immediately identify ambiguous language in the protocol, refine definitions, and append concrete examples to the screening guide prior to the commencement of formal, single-reviewer screening. Following the pilot test, titles and abstracts were screened by the first author for assessment against the inclusion criteria. Potentially relevant sources were retrieved in full. Records that do not contain an abstract (title only) were automatically passed on to full text screening. Abstracts without full texts were also considered for relevance. The full texts of selected citations were assessed in detail against the inclusion criteria by two independent reviewers blinded to each other's decision. Results were then compared and in the event of discrepancies, a consensus reached through discussion. To ensure accuracy in evidence synthesis and prevent duplication of findings, overlapping sources arising from multiple reports of the same study, cohort, or screening programme were collated and linked together, so that each study is the unit of interest in the review, rather than each individual report. Overlapping sources were identified by cross-referencing trial registration numbers; study characteristics e.g. author list, specific geographic locations, intervention protocols; and grant or funding codes. Data from papers included in the scoping review was charted by two independent reviewers using a data extraction form. The extracted data includes details about the authors, year of publication, participants, study design, screening modality and duration, setting, screening guidelines or eligibility criteria adopted, recruitment strategy, and measured outcomes. During data extraction, a single, unified extraction form was completed for each distinct study, synthesizing unique data points across all associated publications (e.g., long-term follow-up data or secondary outcomes). Duplicate findings were explicitly documented and excluded from counting twice. In the final report and PRISMA flow diagram, both the total number of unique studies and the total number of corresponding publications are clearly delineated. A critical appraisal of included studies was conducted using the validated Mixed Methods Appraisal Tool (MMAT) version 2018 (26). For the qualitative synthesis of the included studies, a thematic analysis was conducted following the Braun and Clarke (27) framework. In line with the objective of this study, an inductive approach to coding and theme development was employed, allowing concepts to emerge organically. The first author independently familiarized the text to generate initial codes which were then clustered to identify broader themes. Following a review by the team, these themes were refined and ultimately defined to ensure coherence. Data management and analysis were conducted using MAXQDA software (version 24) (28).

## Results

**Figure 1:**
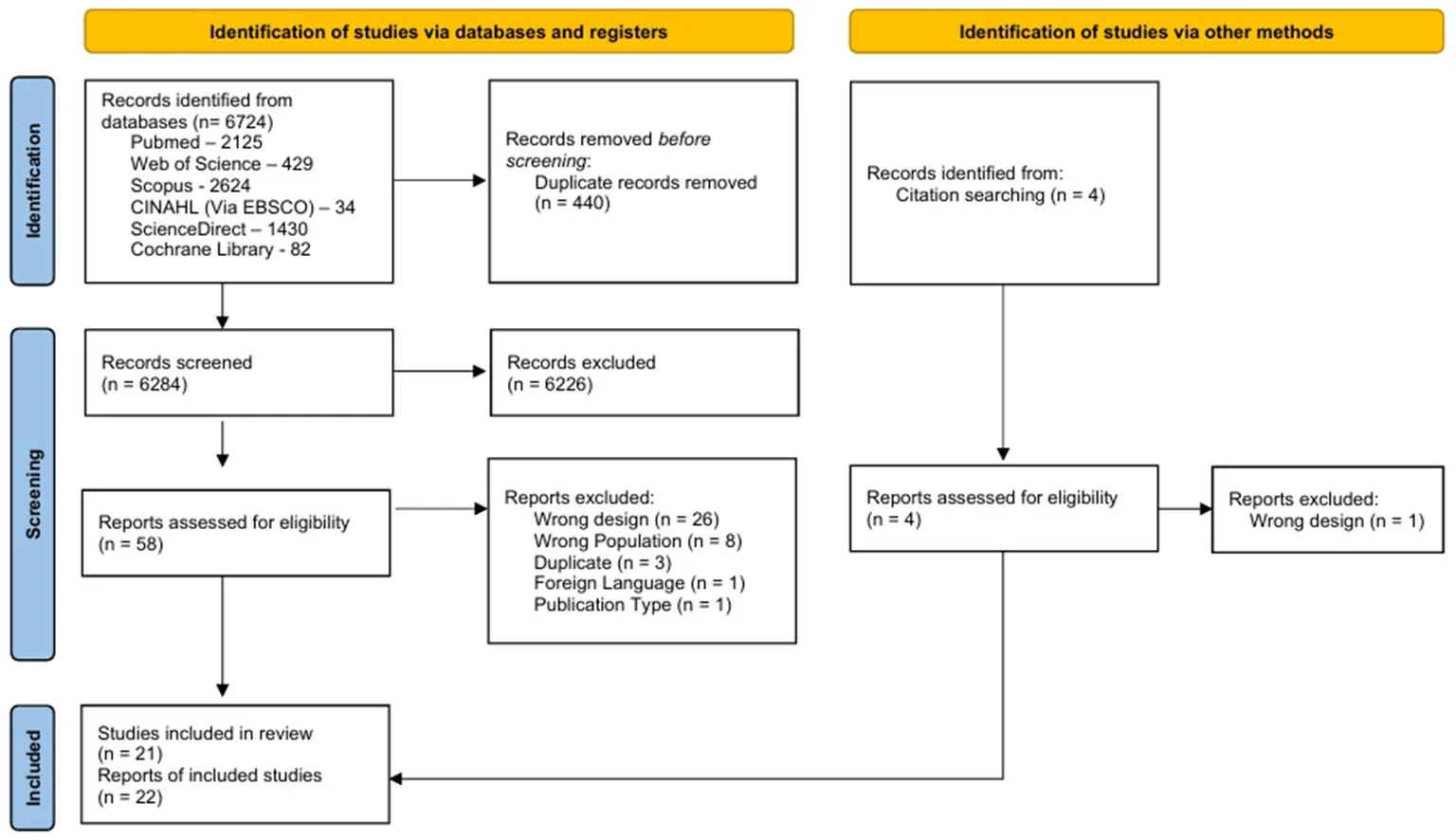
PRISMA flow diagram showing the selection of sources of evidence.

The systematic literature search of six databases; PubMed, Web of Science, Scopus, CINAHL (via EBSCO), ScienceDirect and Cochrane Library, identified 6,724 records, of which 6,284 remained after removal of duplicates. Following title and abstract screening, 58 reports were assessed for eligibility, in addition to 4 reports identified via reference search. A total of 21 studies (29–49) met the inclusion criteria and were included in the review. The most common reasons for full-text exclusion were wrong design (*n* = 26) and wrong population (*n* = 8). The literature selection process is illustrated in the PRISMA flow diagram (Figure 1).

### Characteristics of included studies

The 21 included studies comprised of 19 peer-reviewed full text journal publications, and two conference abstracts (30, 34). In one study, there was complementary updated information in conference proceedings (35, 50). The included sources of evidence are mainly observational studies, including prospective and retrospective cohort studies, cross-sectional studies, and screening program evaluations conducted in the following countries: USA (5), Italy (3), Germany (2), China (2), South Korea (2), Japan (2), Portugal (1), France (1), Iran (1), Egypt (1), and Australia (1). A detailed characteristics of the included studies is presented in Table 3, and their quality assessment shown in Supplementary material 1. Of the 21 included studies, 8 were rated as good quality and 13 as moderate quality, with common limitations including selection bias, limited representativeness, and inadequate control of confounding factors. Nevertheless, the overall evidence base was considered sufficiently robust to support the review findings.

**Table 3:** Characteristics of included studies.

References	Country	Setting	Population (and numbers screened)	Study design	Intervention (and duration)	Eligibility criteria/guideline			Outcome	Recruitment strategy
						Age	Smoking history	Others		
Shah et al. (29)	USA	Emergency Services	Firefighters who are members of the Chicago Fire Department Local 2 Union (*n* = 1,347)	Prospective cohort study	LDCT (April 2022–June 2023)	–	–	• No minimum service requirements. • Exclusion criteria: having a chest CT within the past year. • Continued screening per the 2021 USPSTF LCS guidelines recommended, if a nodule was detected.	Prevalence of Lung RADS category 3 or 4 (high–risk) nodules	Not reported
Grolleau et al. (30)	France	Healthcare	Employees at Lyon University Hospital (LUH) (*n* = 144)	Prospective implementation study	LDCT (Sept. 2022–May 2024)	50–75 years	Smoking more than 15 cigarette/day for 25 years or more than 10 cigarettes/day for 30 years; being current or former smokers who quit since less than 15 years	• French LCS guidelines. • or having a PLCO_m2012_ score ≥ 1.51%; or both	Participation rate	Combination of info displays (flyers, posters); e–mails; and face–to–face interaction through department heads, occupational medicine, and specially trained nurses
Kim et al. (31)	South Korea	Hospitality	Food service workers (*n* = 203)	Cross–sectional study	LDCT (June 2022–Aug. 2022)	≥ 55 years	–	• Had been working for ≥ 10 years • No history of tuberculosis, asthma, or other cancer before screening	Lung–RADS distribution	Not reported
Cabral et al. (32)	Portugal	Mining	A subgroup of former miners of the Uranium National Company (*n* = 66)	Retrospective cohort study	LDCT ^a^ (In 2015)	–	A smoking load greater than 20 pack–years	Exposure to uranium	Prevalence of pulmonary nodules; Lung–RADS distribution	Not reported
Brims et al. (33)	Australia	Diverse^*^	Participants in the Western Australia Asbestos Review Program (*n* = 1,743)	Prospective Cohort study	LDCT (August 2012–August 2017)	–	–	Participants must have had a minimum of 3 months of occupational asbestos exposure and/or radiographically confirmed pleural plaques to participate in the program	Incidence rate of lung cancer	Primary care
Heidrich et al. (34)	Germany	Diverse^*^	German former workers with occupational asbestos exposure (OAE) of ≥ 10 years with onset before 1985 (*n* = 9,277)	Program evaluation	LDCT ^a^	≥55 years	smoking history of ≥30 pack years	Occupational asbestos exposure (OAE) of ≥10 years with onset before 1985	Participation rates and detection rate of lung cancer	Not reported
					Evaluation period: 2014–2021 (Program ongoing)					
Chen et al. (35); Liang et al. (50)	China	Healthcare	Employees of Guangdong Provincial People's Hospital Guangzhou China (*n* = 2,633)	Prospective cohort study	LDCT (Jan. 2019–March 2024)	≥40 years old	–	–	Detection rate of pulmonary nodules and incidence rate of lung cancer	Not reported
Anzai et al. (36)	Japan	Engineering	Employees of Hamamatsu Photonics Engineering Company Japan (*n* = 1,213)	Prospective cohort study	Biomarker + Chest *X*–ray (2009–2019)	>40 years	–	–	• Cancer detection rate • Cancer–related mortality • Cancer–related Health care cost	Not reported
Chung et al. (37)	South Korea	Shipbuilding	Shipyard workers, who underwent health examinations with LDCT (*n* = 6,326)	Retrospective cohort study	LDCT (Jan. 2010 – Dec. 2018)	–	–	• Inclusion Criteria: unclear	Lung RADS distribution; prevalence of Lung–RADS category ≥ 3; detection rate of lung cancer	Not reported
								• Exclusion criteria: female workers (due to their small number); missing information on job type, medical history, and smoking history; never undergone LDCT; and diagnosed with lung cancer before 2010		
Zhang et al. (38)	China	Healthcare	Employees (including retired) from 6 hospitals in different regions of China (*n* = 8,392)	Retrospective cohort study	LDCT (2012–2018)	–	–	Inclusion Criteria: unclear	Detection rate of lung cancer	Not reported
Billatos et al. (39)	USA	Armed Forces	Veterans	Prospective cohort study	Biomarkers + LDCT	• DECAMP 1: ≥45 years	• DECAMP 1: Current or former cigarette smoker with ≥20 pack–year exposure	–	Prevalence of lung cancer; Incidence of lung cancer	Specialty clinics in pulmonary, thoracic surgery and thoracic oncology
			DECAMP 1: (*n* = 500 planned, 489 actual)							
					• DECAMP 1: 2013–2022 (completed)	• DECAMP 2: 50–79 years				
			• DECAMP 2: (*n* = 800 planned, 665 recruited as of 2024)							
							• DECAMP 2: Current or former cigarette smoker (≥10 cigarettes/day for at least 25 years' duration for current smokers, or ≥ 20 pack years for former smokers who quit 20 years ago or less)			
					• DECAMP 2: 09.2011–12.2027 (ongoing)					
Welch et al. (40)	USA	Construction	Workers who had participated in a BTMed screening from 2011 to 2016 and were invited to participate in the Early Lung Cancer Detection (ELCD) Program (*n* = 1,290)	Prospective cohort Study	LDCT ^b^ (2011–2016)	55–74 years	Current smokers with at least 30 pack–years or those who have quit within the past 15 years	• Formerly NCCN (V.2012); Since 2014 NCCN Guideline (Version 1.2014) • Additional requirement of 5 years of work in the construction industry or 5 years of work in a job with exposures to asbestos, silica, beryllium, chromium, radiation or welding	Detection rate of lung cancer; Physical and psychosocial status	Not reported
Markowitz et al. (41)	USA	Construction	Former nuclear weapons workers in 9 non–metropolitan US communities from 2000 −2013 (*n* = 7,189)	Prospective cohort study	LDCT ^b^ (2000–2013)	50 years or older (no upper age limit)	Had smoked for 1 year or longer	Occupation; radiographic asbestos–related fibrosis; and a positive beryllium lymphocyte proliferation test	Detection rate of lung cancer; and Screening Yield per risk factor	Not reported
Barbone et al. (42)	Italy	Construction	Former workers enrolled in the Monfalcone Occupational Health surveillance program for asbestos exposure and resident in the FVG region in early 2002 (*n* = 926)	Prospective cohort study	LDCT (Feb. 2002–Oct. 2003)	40–75 years	Both smokers and non–smokers were eligible	Definite exposure to asbestos, no previous cancer (except non–melanoma skin cancer), no severe comorbidities, no clinical suspicion of lung cancer or MNP and no chest CT scan during the previous 2 years	Standardized incidence ratios (SIRs) and standardized mortality ratios (SMRs)	Not reported
Abtahi et al. (43)	Iran	Glass wool manufacturing	Employees of a glass wool company (*n* = 145)	Analytical Cross–sectional study	Biomarkers (Serum CEA and CYFRA 21–1 Levels)	No age	No smoking history limitation	Only employees working 8 hours a day	Concentration levels of serum CEA and CYFRA 21–1	Not reported
Kato et al. (44)	Japan	Diverse^*^	Workers with histories of asbestos exposure enrolled between 2010 and 2012 (*n* = 2,132)	Descriptive Cross–sectional study	LDCT (2010–2012)	–	–	• Engagement in asbestos–product manufacturing for more than 1 year • Engagement in other industries related to asbestos exposure for more than 10 years, or • Engagement in industries related to asbestos exposure and demonstrated pleural plaques on chest X–ray or CT (regardless of the duration of asbestos exposure)	Prevalence of lung cancer and malignant pleural mesothelioma (MPM)	Not reported
Smargiassi et al. (45)	Italy	Diverse^*^	Former male asbestos–exposed workers in the Campania Region of Italy (*n* = 59)	Descriptive Cross–sectional study	LDCT + Chest ultrasonography (Nov. 2015 –Feb. 2016)	–	–	Occupational exposure to asbestos fibers before 1992	Detection rate of pathological findings like Pleural thickening, peripheral lung consolidation, pulmonary asbestosis, etc.	E–mails only
Gaballah et al. (46)	Egypt	Wood	Male workers in the carpentry section of wooden furniture manufacturer in an Egyptian modernized industrial factory located in Greater Cairo (*n* = 86)	Analytical Cross–sectional study	Sputum PCR –	–	Excluded moderate to heavy smokers (Smoking Index ≥ 20 pack years)	Inclusion Criteria: • Wood dust exposure at the workplace for more than 5 years	Frequency of chromosomal aberrations (CA) and sister chromatid exchanges (SCE) in peripheral blood lymphocytes (PBL); Superoxide dismutase (SOD) and glutathione peroxidase (GPx) enzymes levels	Not reported
								Exclusion Criteria: • Exposures with other chemical exposures at workplace in addition to wood dust. • Present history of hypertension or diabetes. • Familial history of any type of cancer. • Abnormal liver function tests.		
Okereke et al. (47)	USA	Armed Forces	US Veterans at the Providence VAMC in Providence, Rhode Island (*n* = 1,832)	Retrospective review	LDCT ^a^ (Dec. 2013–Dec. 2014)	55–74 years	Current smokers or quit within the past 15 years; and had at least a 30–pack–year smoking history	–	Detection rate of lung cancer	Primary care
Felten et al. (48)	Germany	Power	Asbestos–exposed employees of a major provider of electrical power in Germany (*n* = 187)	Prospective cohort study	LDCT and sputum cytology (Sept. 2002–July 2006)	≤75 years old	–	Signed declaration of past contact with asbestos	Detection rate of lung cancer; Pathological findings	Not reported
Mastrangelo et al. (49)	Italy	Diverse^*^	• Categories of workers with the highest risk of asbestos exposure in the Veneto Region of Italy (*n* = 1,165)	Program evaluation	LDCT ^b^	55–59 years	–	Only males included	• Detection rate of lung cancer • Incidence rate of lung cancer • Cost • Radiation dose	Invitation letters to participants and their family physicians
					• 2000–2005 (LCS project)					
					• 2006–2011 (on–demand health surveillance)					
			• b) All workers, whatever the previous level of asbestos exposure, as part of an on–demand health surveillance (*n* = 3,149)							

^a^Including a shared decision–making.

^b^Including a smoking cessation intervention.

^*^Diverse refers to when participants of a LCS program are recruited from a combination of different occupational settings or industries, particularly those characterized with Asbestos Exposure.

#### Occupational settings and study populations

The study populations, defined by occupational setting, included hospital employees (30, 35, 38), firefighters (29), food service workers (31), miners (32), engineering company employees (36), shipyard workers (37), veterans (39, 47), construction workers (40–42), glass wool company employees (43), carpenters (46) and electrical power employees (48). Seven studies specifically targeted asbestos-exposed populations (33, 34, 42, 44, 45, 48, 49), while one targeted a uranium-exposed population (32). **Six** studies involved participants of existing occupational health surveillance programs, including; US Department of Energy (DOE) Building Trades National Medical Screening Program (BTMed) (40); Health Intervention Program for former employees of the Uranium National Company (UNC) and their families in Portugal (32); a voluntary union-sponsored multisite occupational medicine screening program for former US DOE workers (41); Monfalcone Occupational Health surveillance program for asbestos exposure in Italy (42); Western Australia Asbestos Review Program (ARP) (33); and a LCS of formerly asbestos-exposed workers in Germany (EVA-LCS) (34). There was a wide variation in sample sizes, with the least recorded sample size being 59 (45) and the highest 9,277 participants (34).

#### Eligibility criteria and risk assessment approaches

Eligibility criteria for participation in LCS activities varied considerably across the evidence, with notable variations even among similar occupational group (Table 3). Twelve publications considered age as an eligibility criterion, with three making it the only criterion (35, 36, 49). Seven considered smoking history (30, 32, 34, 39–41, 47). Twelve considered occupational history, focusing specifically on minimum duration of service (31, 43), occupational risk factor exposure (32–34, 42, 44–46, 48), or either of both (40, 41). Five considered medical history (31, 33, 34, 41, 42). One used a country guideline (France) that considered in addition to age and smoking history, the PLCO_m2012_ risk score (30). One study used the NCCN guideline (40). The eligibility criteria in two studies were unclear (37, 38). Most of the studies that were conducted in Europe and North America considered eligible, current or former smokers aged 50–80 years. In contrast, the studies from Asia included younger participants, often below 50 years of age, and notably incorporated never-smokers into the screening cohorts (35, 38). The methodologies employed to capture and quantify exposure to workplace carcinogens included: anamnestic and self-reported questionnaires (31–33, 35–37, 39–42, 45, 46, 48, 49), semi-quantitative and job-exposure matrices (37, 41, 42, 49), environmental and workplace monitoring (31, 43, 46), personal dosimetry (35, 36), biomarkers (43, 46, 49), clinical and radiological markers (33, 40, 41, 44, 49). There was no direct occupational risk assessment in three studies (29, 30, 38).

#### Recruitment strategies

Recruitment strategies for the LCS or interventions to enhance participation of eligible employees were reported in six of the 21 studies (30, 33, 39, 45, 47, 49). The following individual strategies were identified: e-mails, invitation letters to participants, face-to-face interaction, direct contact by department heads or supervisors, primary care, specialty clinics in pulmonary, thoracic surgery and thoracic oncology, or their combinations (Table 3). Grolleau et al. (30) assessed the effect of the various communication methods on participation rate. A shared decision making (SDM) process was reported in three studies (32, 34, 47).

#### Interventions and screening protocols

The following screening methods were used: LDCT only (29–35, 37, 38, 40–42, 44, 47, 49), LDCT + sputum cytology (48), LDCT + biomarker (39), LDCT + chest ultrasonography (45), biomarker + chest *X*-ray (36), biomarker only (43), and sputum PCR (46). Therefore, 18 studies utilized LDCT for screening-−15 as a standalone method and three in combination with adjuncts. The studies that employed biomarker only and sputum PCR did not directly aim to detect lung cancer, but rather to predict or indicate progression toward lung cancer.

The duration of intervention (including follow-ups) varied across the studies, with the least recorded duration being 3 months and the highest 13 years for already concluded studies (Table 3). One of the studies is still ongoing, expected to be completed in 2027 with a 16-year intervention duration (39). Several studies tracked cohorts over multi-year intervals to account for long latency periods e.g. Brims et al. (33) and Felten et al. (48), with a mean time since first exposure (TSFE) to asbestos of 51.7 years and 40.4 years respectively (33, 48), or those featuring continuous annual screening rounds to catch delayed incidence peaks decades after exposure e.g. Mastrangelo et al. (49). In contrast, some cross-sectional and pilot studies utilize single baseline scans or brief multi-month windows, managing latency by enforcing strict eligibility thresholds such as specific age minimums, historical sector employment, or years of workplace exposure e.g. Kim et al. (31), Kato et al. (44) and Smargiassi et al. (45) utilized cross-sectional designs where participants received a single baseline scan (31, 44, 45). Kim et al. (31) conducted LDCT screening over a brief three-month window in 2022, managing latency by requiring participants to have ≥10 years of food service experience or an age ≥ 55 years (31). Similarly, Kato et al. (44) included ≥10 years work experience in industries related to asbestos exposure as an eligibility criterion (44). To capture long-term risks, Smargiassi et al. (45) evaluated a cohort with a mean of 44.1 years since initial asbestos exposure (45). Institutional programs incorporated systematic, multi-year annual rescreening protocols to consistently manage long-term risks (34), Markowitz et al. (41). The LCS for formerly asbestos-exposed employees (EVA-LCS) in Germany is an ongoing program since 2014 (34). Three studies did not specify the duration of intervention (32, 43, 46). In three studies, smoking cessation intervention was offered to participants who were active smokers (40, 41, 49).

### Screening outcomes

The most reported outcome among the included studies was lung cancer burden, measured by detection rate (34, 36–38, 40, 41, 47–49), incidence rate (33, 35, 39, 42, 49), and prevalence rate (39, 44) (Table 3). Other outcomes included Lung RADS distribution (29, 31, 32, 37), detection rate of other pathological findings (45, 48), screening participation (30, 34), cancer-specific mortality (36, 42), healthcare costs (36, 49) and radiation dose (49). The two studies that used biomarkers only and sputum PCR reported biomarker serum concentration levels as their outcomes (43, 46). False-positive results were evaluated in some studies (29, 37, 47, 48) and some systematically evaluated physical procedural complications from follow-up diagnostic localization or biopsy procedures within their cohorts (29, 40).

### Thematic synthesis of included studies

All 21 included studies contributed to the thematic synthesis, yielding the following overarching themes: (1) Occupational exposure as lung cancer risk factors and limitations in current LCS guidelines, (2) Lung cancer burden across diverse occupational groups, (3) Benefits and performance of LDCT screening in occupational cohorts, (4) Tools and multimodal strategies to enhance LDCT, and (5) Implementation, participation and program feasibility. A mapping of the evidence is shown in Table 4.

**Table 4:** Mapping the evidence.

Theme	Description	Key evidence	Representative studies
Occupational exposures as lung cancer risk factors and limitations in current LCS guidelines	To identify how specific occupational exposures contribute to lung cancer risk	• Firefighting exposure independently increases lung cancer risk • Cooking–oil fumes are associated with higher Lung–RADS categories • Wood–dust exposure linked to increased radiologic abnormalities and cancer markers • Heavy exposure to welding fumes, chromium, and nickel is independently associated with higher prevalence of positive screening findings • Smoking–based criteria fail to capture many high–risk asbestos–exposed workers • Combined smoking + occupational exposures dramatically elevate risk	Shah et al. (29); Kato et al. (44); Kim et al. (31); Chung et al. (37); Gaballah et al. (46); Abtahi et al. (43); Welch et al. (40); Brims et al. (33); Markowitz et al. (41)
Lung–cancer burden in diverse occupational groups	To assess incidence and prevalence rates of lung cancer	• Hospital employees show higher lung cancer incidence than general population • Employer–driven screening detects more early lung cancers than expected	Chen et al. (35); Zhang et al. (38); Anzai et al. (36); Okereke et al. (47)
Benefits and performance of LDCT screening in occupational cohorts	To evaluate early–detection performance and mortality outcomes of LDCT in occupational groups	• LDCT detects cancers at earlier stages in high–risk workers • Mortality reductions reported in asbestos cohorts undergoing LDCT • LDCT more effective for early detection than traditional surveillance methods	Cabral et al. (32); Zhang et al. (38); Brims et al. (33); Barbone et al. (42); Mastrangelo et al. (49)
Tools and multimodal strategies to enhance LDCT	To assess whether biomarkers, cytology, or imaging adjuncts improve screening performance	• DECAMP integrates biomarkers + LDCT to enhance early detection • Glass wool workers show elevated tumor markers indicating added risk stratification utility • Chest ultrasonography feasible for monitoring asbestos–related pleural disease • Sputum cytology has low sensitivity but marginally improves detection when added to LDCT	Billatos et al. (39); Abtahi et al. (43); Smargiassi et al. (45); Felten et al. (48)
Implementation, participation and program feasibility	Understanding barriers and facilitators to screening uptake among workers	• Studies highlight the need to explore more measures to improve participation of workers in a LCS program • Tailored communication and centralized scheduling improve participation • Clinical reminders in electronic health records are valuable for monitoring participation	Grolleau et al. (30); Heidrich et al. (34); Zhang et al. (38); Cabral et al. (32); Okereke et al. (47)

#### Occupational exposure as lung cancer risk factors and limitations in current LCS guidelines

A substantial body of evidence across the included studies demonstrates that occupational exposures are major determinants of lung-cancer risk yet remain insufficiently captured by current screening eligibility criteria. Shah et al. (29) reported that among 1,347 screened firefighters, 41 (3.0%) had Lung-RADS 3–4 nodules. Brims et al. (33) and Kato et al. (44) reported 26 (1.5%) and 45 (2.1%) detected lung cancers across 1,743 and 2,132 asbestos-exposed workers respectively. Kim et al. (31) found a 10.2% Lung-RADS–positive rate among school cafeteria workers exposed to cooking-oil fumes. In their evaluation of 6,326 Korean shipyard workers, Chung et al. (37) demonstrated that heavy exposure to occupational carcinogens like welding fumes, chromium, and nickel was independently associated with a significantly higher prevalence of positive screening findings. Gaballah et al. (46) and Abtahi et al. (43) documented early detection signals and elevated tumor markers among wood workers and those exposed to respirable synthetic vitreous fibers (SVF), respectively. Welch et al. (40) similarly observed increased radiologic abnormalities among mixed-exposure construction cohorts.

Simultaneously, several studies highlighted limitations in existing LCS guidelines for detecting lung cancer in worker populations. Approximately 20 of the 41 firefighters with high-risk nodules in Shah et al. (29) were never-smokers and therefore ineligible for screening. Only six were eligible under USPSTF criteria, meaning that 35 positive screens would have been missed. In Markowitz et al. (41), one-third of detected lung cancers (24/69) occurred among former nuclear weapons workers who had quit smoking ≥15 years earlier and would not have qualified under NLST criteria. Brims et al. (33) similarly showed that smoking-based criteria fail to identify many asbestos-exposed workers who develop lung cancer. Overall, findings strongly indicate that occupational exposure is a critical risk factor inadequately addressed in current LDCT eligibility frameworks. Evidence consistently supports the development of eligibility criteria that explicitly incorporate occupational exposures and broadened smoking and age thresholds to improve early detection among high-risk worker populations.

#### Lung cancer burden across diverse occupational groups

A measurable lung cancer burden was observed not only among workers with recognized high-risk occupational exposures but also among groups with low or no clearly defined occupational carcinogenic exposure. Chen et al. (35) detected suspicious nodules in 9.1% and confirmed lung cancer in 4.0% among 2,552 hospital employees. Similarly, Zhang et al. (38) detected 179 (2.1%) lung cancers among 8,392 hospital employees, particularly in young, female, and non-smoking staff during routine health examinations. An employer-initiated screening program in Japan identified 14 lung cancers (0.8%) among corporate employees in roles not associated with known carcinogenic exposure (36). Okereke et al. (47) detected 27 cancers among 2,106 screened veterans whose risk was primarily driven by smoking rather than occupational carcinogens. Overall, the evidence demonstrates that lung cancer burden extends beyond traditionally recognized high-risk occupations to include groups with diffuse, low-dose, or indiscernible exposures. This pattern suggests that LCS may hold value across a wider range of occupational settings than conventional exposure-based models assume.

#### Benefits and performance of LDCT screening in occupational cohorts

Studies assessing the benefits of LDCT screening in occupational settings consistently showed favorable early-detection performance. Cabral et al. (32) reported that LDCT screening among high-risk Portuguese workers detected early-stage cancers at a meaningful rate while maintaining low false-positive proportions. Several studies among asbestos-exposed cohorts reinforced this benefit: Brims et al. (33) reported that LDCT yields are high even among workers who would not qualify under smoking-based criteria. Similarly, Barbone et al. (42) demonstrated significant mortality reduction associated with LDCT screening in asbestos-exposed italian workers, while Mastrangelo et al. (49) found that post-occupational surveillance with LDCT improved early detection compared with previous radiographic follow-up models. Zhang et al. (38) reported that 95% of the 179 detected lung cancer cases from LDCT were stage-1. Together, these studies support LDCT as an effective tool for early detection and potentially mortality reduction in occupational groups. However, several studies explicitly evaluated the physical and psychological burdens associated with introducing LDCT screening to occupationally exposed workforces. Welch et al. (40), Brims et al. (33), Mastrangelo et al. (49) and Markowitz et al. (41) tracked ionizing radiation burdens, recording very low to median dose of 0.2 – 1.36 mSv which fell within acceptable safety boundaries. However, Mastrangelo et al. (49) reported a staggering 220 mSv for each screen-detected lung cancer case. Assessing psychological distress, Felten et al. (48) documented severe emotional harm, highlighting a participant who required psychiatric hospitalization for depression after receiving notice of a suspicious lung nodule, leading the authors to mandate that screening programs integrate specialized psychological support networks. Chen et al. (35) and Cabral et al. (32) likewise discussed the elevated psychological burden and patient anxiety triggered by high false-positive rates. Conversely, Welch et al. (40) systematically tracked mental health using the SF-12 questionnaire and found no significant negative psychosocial impacts between nodule-free participants and those tracking with indeterminate findings.

#### Tools and multimodal strategies to enhance LDCT

Whilst LDCT remains the only method currently recommended for LCS per USPSTF and NCCN guidelines, some of the included studies tested adjuncts intended to improve risk stratification, reduce false positives, or guide follow-up. Billatos et al. (39) described the DECAMP protocol, which combines biomarkers with LDCT to better discriminate malignant from benign nodules. Smargiassi et al. (45) described chest ultrasonography as a feasible adjunct for pleural disease surveillance, and Felten et al. (48) evaluated sputum cytology combined with LDCT (limited additional sensitivity). Abtahi et al. (43) observed elevated tumor markers (CEA, CA-125, CYFRA21-1) among glass wool workers, suggesting potential utility for biomarker-based risk refinement. The studies that employed biomarker only and sputum PCR did not directly target lung cancer detection but rather aimed to predict or indicate progression toward the disease. Overall, adjunctive strategies appear promising but largely remain in feasibility/early-validation stages. None have received endorsement from major bodies such as the USPSTF or the NCCN for routine clinical use.

#### Implementation, participation and program feasibility

Participation-focused studies were few but informative. Grolleau et al. (30), reporting from the first round of the ILYAD program in France, showed a participation rate of 17.2% in hospital workers. Heidrich et al. (34) in their evaluation of the EVA-LCS program reported a participation rate of 40.7 % among former asbestos exposed workers. Zhang et al. (38) reported an overall LDCT participation rate of 53.5%. The studies highlight the need to explore more measures to improve participation of workers in a LCS program. In their survey of employers in Japan, Minamitani et al. (51) demonstrated that employers' interest in cancer control, history of cancer screening and/or cancer, policies for implementation of cancer screening and support measures for balancing treatment and work, plays a big role in the implementation of LCS in occupational setting. Okereke et al. (47) reported that the introduction of LDCT at a Veteran Affairs medical center produced moderate early uptake and acceptable follow-up adherence. The use of clinical reminders embedded in electronic health record was valuable in reminding (47) as well as in assessing the rate of declining participation in LCS and factors associated with it (52). In the studies by Cabral et al. (32), Heidrich et al. (34), and Okereke et al. (47), a shared decision making (SDM) process was carried out, which in addition to patient empowerment, also improves participation and retention of eligible employees in LCS programs.

## Discussion

This scoping review maps the available evidence on LCS in occupational settings. Following the systematic literature search in six databases, 21 studies were included. All were published in English and conducted across eleven countries. They covered diverse occupational settings and worker populations, including those with known occupational exposures and those with no discernible occupational risks. LDCT was the predominant screening modality, employed in 18 of the 21 studies. Some adjunct tools were also identified, primarily used to improve risk stratification. There were notable variations in eligibility criteria, involving factors like age, smoking history, occupational history, and medical history. The following themes were identified across the studies: Occupational exposure as lung cancer risk factors and limitations in current LCS guidelines; Lung cancer burden in occupational groups; Benefits and performance of LDCT screening in occupational cohorts; Tools and multimodal strategies to enhance LDCT; and Implementation, participation and program feasibility.

The high proportion of screen-detected high-risk nodules among never-smokers in some occupational cohorts (notably firefighters) was unexpected, thus underscoring limitations in current LCS guidelines in relation to early lung cancer detection among worker populations. This was more evident in studies from Asia that included younger participants, often below 50 years of age, and notably incorporated never-smokers into screening cohorts (35, 38). This suggests that occupational exposures may create risk profiles discordant with smoking-based criteria (NLST, USPSTF) or that epidemiological differences in lung cancer risk exist across regions. The former supports considering exposure-inclusive or hybrid eligibility models when screening worker populations. This does not directly contradict population guidelines (which were developed for general populations and smokers) but indicates that policy adaptations may be justified for defined occupational cohorts. The latter would demonstrate a need to tailor lung cancer guidelines to the specific risk profiles of regional populations, as guidelines can directly inform eligibility criteria adoption in occupational LCS in these regions. Therefore, in LCS of worker populations, eligibility criteria that incorporate a broader range of occupational exposures to workplace lung carcinogens, along with expanded age and smoking history thresholds, may improve early lung cancer detection outcomes. In all, a one-size-fits-all approach to screening may overlook significant at-risk subgroups, particularly in regions where non-smoking-related lung cancer is increasingly prevalent (53). Furthermore, a recent study by Oddone et al. (54) exploring the relationship between occupational exposure and mutational burden of lung cancer suggests that a worker's occupational history may provide valuable prognostic information.

Crucially, evidence on LCS in occupational settings remains limited, with the existing literature consisting largely of observational studies. There is a relative paucity of robust, prospective, randomized controlled studies (RCTs) on LCS in occupational cohorts that can provide stronger causal inference in specific exposure groups, as well as on long-term effects, health equity, and cost-effectiveness. However, the largest gap lies in the lack of standardized screening protocols, occupational exposure metrics and reporting on screening processes and outcomes. Robust evaluation studies are essential to validate and optimize occupational lung cancer screening. As demonstrated by Greiner et al. (55), comprehensive frameworks leverage large-scale, nationwide cohorts to establish quality assurance indicators, track long-term harm-to-benefit metrics, as well as assess participant psychological distress. Hence, large validation studies for adjunctive tools (including Artificial Intelligence), implementation studies in diverse worker populations, particularly on strategies to maximize equitable uptake (addressing shift work, confidentiality), as well as studies on psychosocial outcomes (anxiety, worry, etc) are needed. Research is also limited in non-industrial occupational groups and in low- and middle-income countries.

### Strengths and limitations

A major strength of this review is its comprehensive, systematic search strategy across multiple databases. The broad scope allowed identification of diverse research directions. Additionally, a critical appraisal of the included studies provides insight into the methodological quality and potential risk of bias of the evidence base. Most of the studies were rated as moderate due to methodological limitations like selection bias, limited representativeness, and inadequate control of confounding factors. These limitations should be taken into account when interpreting the results and considering the generalisability of the conclusions. While this appraisal enhances the interpretability of the findings, it was not used to exclude studies and should be considered as supplementary to the evidence mapping approach. The heterogeneity of study designs made direct comparisons difficult. Also, the review considered only publications made in English and German, potentially excluding relevant studies in other languages.

### Implications for research and practice

The findings highlight the need for standardized implementation protocols and adherence to established reporting guidelines. To facilitate effective replication of LCS programs across diverse occupational settings, studies should provide comprehensive reporting of key implementation components, including recruitment strategies, participation rates, and cohort retention methods. Such transparency enables researchers and policymakers to evaluate the feasibility, scalability, uptake, and sustainability of LCS programs across different worker populations, especially considering the heterogeneity in study designs, populations, and screening protocols. Future studies should also employ rigorous methodological approaches and follow reporting guidelines such as STROBE Statement (56) or CONSORT (57), where appropriate, to strengthen the evidence base for occupational LCS.

The review suggests the potential to integrate LDCT screening into existing occupational health programs, using mobile/on-site screening to maximize reach and convenience, paired with tailored education to improve informed uptake.

## Conclusion

Occupational settings present significant yet under-recognized opportunities for the early detection of lung cancer. With screening efforts rightly focused on workers at clearly elevated exposure-related risk, the findings demonstrate potential benefits for individuals with low or uncharacterized occupational exposures. However, the evidence base remains limited and fragmented, with significant gaps in implementation protocols and reporting standards. Addressing these deficiencies requires a concerted research effort toward further robust prospective studies, specifically RCTs, supported by standardized frameworks. Such evidence will be essential to ensure the seamless translation of occupational LCS from an exploratory workplace initiative into a reproducible, scalable public health intervention across diverse occupational settings.

## Data Availability

The original contributions presented in the study are included in the article/Supplementary material, further inquiries can be directed to the corresponding author.
