# Supplementary material for: Lung cancer screening in occupational settings: a scoping review
Source: Front Public Health. 2026 Aug 27;14:1888829. doi: 10.3389/fpubh.2026.1888829 (PMC13560921; doi:10.3389/fpubh.2026.1888829)
Supplement: Supplementary file 1 [file Table_1.docx]

**Supplementary Material 1: Critical Appraisal of included studies using the Mixed Methods Appraisal Tool (version 18)**

| Author(s) | Study design | Methodological quality criteria | Responses | Quality Assessment |
| --- | --- | --- | --- | --- |
| Shah et al. (29) | Prospective cohort study | S1. Are there clear research questions? | Yes. The study clearly aims to evaluate whether firefighting is associated with high-risk lung nodules on LDCT and if standard guidelines miss these high-risk individuals. | Moderate |
|  |  | S2. Do the collected data allow to address the research questions? | Yes. The authors gathered demographic, smoking history, and radiographic (Lung-RADS) data from 1,347 firefighters to directly calculate nodule prevalence and criteria eligibility. |  |
|  |  | 3.1. Are the participants representative of the target population? | Partially. Though the study included a fairly large sample of smoking and non-smoking firefighters, there is a risk of selection bias, limiting generalization |  |
|  |  | 3.2. Are measurements appropriate regarding both the outcome and intervention (or exposure)? | Yes. Exposure (firefighting) was clearly defined based on occupational history, and outcomes (lung cancer detection) were assessed using standardized LDCT and Lung-RADS criteria, supporting measurement validity. |  |
|  |  | 3.3. Are there complete outcome data? | Yes. Screening outcomes, including eligibility rates and cancer detection, were clearly reported for the study population, with no indication of substantial missing outcome data. |  |
|  |  | 3.4. Are the confounders accounted for in the design and analysis? | No. Key confounders like age or smoking history were not accounted for. |  |
|  |  | 3.5. During the study period, is the intervention administered (or exposure occurred) as intended? | Yes. LDCT was conducted for all eligible firefighters. |  |
| Grolleau et al. (30) | Prospective implementation study | S1. Are there clear research questions? | Yes. The study clearly seeks to evaluate which communication methods best optimize participation in an LCS program. | Moderate |
|  |  | S2. Do the collected data allow to address the research questions? | Yes. The data tracks the number of inclusions per communication period and overall participation rates. |  |
|  |  | 4.1. Is the sampling strategy relevant to the research question? | Yes. The study targets the total eligible population within a specific institution to test recruitment efficacy |  |
|  |  | 4.2. Is the sample representative of the target population? | Yes. The sample is the entire identified eligible staff at the hospital (838 people) |  |
|  |  | 4.3. Are the measurements appropriate? | Yes. Participation rates and the source of recruitment are standard, appropriate metrics for this type of study. |  |
|  |  | 4.4. Is the risk of non-response bias low? | No. The very low participation rate (17.2%) suggests a high risk of non-response bias. |  |
|  |  | 4.5. Is the statistical analysis appropriate? | Yes. Descriptive statistics are appropriate for study aim. |  |
| Kim et al. (31) | Cross-sectional study | S1. Are there clear research questions? | Yes. The study aims to identify Lung-RADS distribution and related risk factors among cafeteria workers. | Good |
|  |  | S2. Do the collected data allow to address the research questions? | Yes. Data includes LDCT results (Lung-RADS) and exposure variables from questionnaires. |  |
|  |  | 3.1. Are the participants representative of the target population? | Yes. Participants were an occupational group of female school cafeteria workers (cooks/nutritionists) meeting specific age/tenure criteria |  |
|  |  | 3.2. Are measurements appropriate regarding both the outcome and intervention (or exposure)? | Yes. Standardized Lung-RADS criteria was used to measure outcomes and cooking oil fume exposure was assessed systematically (e.g., job tasks/ frequency, and ventilation). |  |
|  |  | 3.3. Are there complete outcome data? | Yes. Lung-RADS distribution among participants was clearly reported, with stratification by exposure group. |  |
|  |  | 3.4. Are the confounders accounted for in the design and analysis? | Yes. Binomial logistic regression analyses were performed, adjusting for relevant confounders. |  |
|  |  | 3.5. During the study period, is the intervention administered (or exposure occurred) as intended? | Yes. Exposure to cooking oil fumes was determined by routine occupational conditions. |  |
| Cabral et al. (32) | Retrospective cohort study | S1. Are there clear research questions? | Yes. The study aims to estimate the benefit of LDCT for lung cancer screening in a high-risk population of former miners. | Moderate |
|  |  | S2. Do the collected data allow to address the research questions? | Yes. Data include initial medical assessments, questionnaires, and LDCT results (nodule classification and follow-up). |  |
|  |  | 3.1. Are the participants representative of the target population? | Partially. The sample consists of high-risk former uranium miners. However, low participation (66/265) limits representativeness. |  |
|  |  | 3.2. Are measurements appropriate regarding both the outcome and intervention (or exposure)? | Yes. LDCT was used for screening and lung nodules classified according to established Fleischner Society guidelines. |  |
|  |  | 3.3. Are there complete outcome data? | Yes. The study accounts for all 66 miners in the second phase and details their specific CT results and follow-up paths. |  |
|  |  | 3.4. Are the confounders accounted for in the design and analysis? | No. Important confounders (e.g. radon exposure) were acknowledged but not controlled. |  |
|  |  | 3.5. During the study period, is the intervention administered (or exposure occurred) as intended? | Yes. LDCT performed using standardized protocol and nodule management based on Fleischner Society guidelines. |  |
| Brims et al. (33) | Prospective Cohort study | S1. Are there clear research questions? | Yes. The study aims to analyze LDCT effectiveness in an asbestos-exposed population and compare eligibility criteria of existing screening programs. | Good |
|  |  | S2. Do the collected data allow to address the research questions? | Yes. Data include 5,702 LDCT scans, lung function, smoking history, and asbestos exposure levels linked to cancer registry data. |  |
|  |  | 3.1. Are the participants representative of the target population? | Yes. The study participants, drawn from the Asbestos Review Program (ARP), included a fairly large sample that is representative of the target population. |  |
|  |  | 3.2. Are measurements appropriate regarding both the outcome and intervention (or exposure)? | Yes. Outcomes (lung cancer detection) were measured using valid modality: a standardized LDCT and cancer registry linkage. Exposure (asbestos) was based on occupational history or self-report. |  |
|  |  | 3.3. Are there complete outcome data? | Yes. Screening outcomes, including detection rates and findings, were clearly reported for participants, with no indication of substantial missing data. |  |
|  |  | 3.4. Are the confounders accounted for in the design and analysis? | Yes. Stratification of variables was applied. |  |
|  |  | 3.5. During the study period, is the intervention administered (or exposure occurred) as intended? | Yes. LDCT imaging followed a standardized protocol with consistent nodule follow-up procedures. Exposure to asbestos was a criterion for inclusion in the study. |  |
| Heidrich et al. (34) | Program evaluation | S1. Are there clear research questions? | Yes. The study aims to evaluate the process and outcomes of the German Social Accident Insurance (DGUV) LCS program using routine data. | Moderate |
|  |  | S2. Do the collected data allow to address the research questions? | Yes. The study uses routine administrative and medical data of eligible persons to assess participation rates, suspicious findings, and cancer detection. |  |
|  |  | 4.1. Is the sampling strategy relevant to address the research question? | Yes. There is a clearly defined inclusion and exclusion criteria, and all eligible persons were centrally identified and invited for screening. |  |
|  |  | 4.2. Is the sample representative of the target population? | Yes. By using official data, the sample accurately reflects the actual target population. However, there was a participation rate of only 40.7%. |  |
|  |  | 4.3. Are the measurements appropriate? | Yes. Key outcomes, including participation rates and LDCT findings, were reported. |  |
|  |  | 4.4. Is the risk of nonresponse bias low? | No. A substantial proportion of eligible individuals did not participate, and no comparison between participants and non-participants is reported. |  |
|  |  | 4.5. Is the statistical analysis appropriate to answer the research question? | Yes. The study primarily used descriptive statistics (participation rates, detection rates), which are appropriate for the study aim. |  |
| Chen et al. (35); Liang et al. (50) | Prospective cohort study | S1. Are there clear research questions? | Yes. Both studies explicitly aim to determine the detection rate and incidence of lung cancer while identifying potential occupational/environmental risk factors. | Good |
|  |  | S2. Do the collected data allow to address the research questions? | Yes. The use of LDCT screening, pathological confirmation, and environmental monitoring directly aligns with the stated objectives. |  |
|  |  | 3.1. Are the participants representative of the target population? | Yes. There is a high participation rate of eligible participants (85.9%), representative of the target population |  |
|  |  | 3.2. Are measurements appropriate regarding both the outcome and intervention (or exposure)? | Yes. Outcomes (lung cancer incidence rate) were measured using a standardized and valid modality (LDCT). Key exposures (e.g., smoking) were derived from health examination data. |  |
|  |  | 3.3. Are there complete outcome data? | Yes. Number of cases and incidence rates were systematically recorded over the follow-up period, with no indication of significant missing outcome data. |  |
|  |  | 3.4. Are the confounders accounted for in the design and analysis? | Partially. While the study examined associations between risk factors and lung cancer, there is no clear evidence of comprehensive multivariable adjustment for key confounders. |  |
|  |  | 3.5. During the study period, is the intervention administered (or exposure occurred) as intended? | Yes. The screening protocol and surgical decision-making followed a consistent workflow |  |
| Anzai et al. (36) | Prospective cohort study | S1. Are there clear research questions? | Yes. The study aims to determine cancer detection, mortality, and financial implications of the screening program. | Moderate |
|  |  | S2. Do the collected data allow to address the research questions? | Yes. Data includes biopsy-proven diagnoses, mortality records, and insurance-based cost tracking. |  |
|  |  | 3.1. Are the participants representative of the target population? | Partially. Consists of a single-company cohort of younger, well-educated, high health literacy participants with a higher male-to-female ratio. While representative of that workforce, there is a limited generalizability to the broader population. |  |
|  |  | 3.2. Are measurements appropriate regarding both the outcome and intervention (or exposure)? | Yes. There are clearly defined outcomes (biopsy-proven cancer, cancer mortality and healthcare costs), with standardized imaging protocol. |  |
|  |  | 3.3. Are there complete outcome data? | Yes. Screening outcomes were clearly reported for participants, with minimal loss to follow-up |  |
|  |  | 3.4. Are the confounders accounted for in the design and analysis? | No. there was no adjustment for key confounders (e.g. Smoking, family history) |  |
|  |  | 3.5. During the study period, is the intervention administered (or exposure occurred) as intended? | Yes. Structured screening every 2 years over 10 years, clear protocol and consistent implementation. However, there is a lack of randomization of health intervention. |  |
| Chung et al. (37) | Retrospective cohort study | S1. Are there clear research questions? | Yes. The study clearly aims to evaluate the distribution of Lung-RADS categories in shipyard workers exposed to nickel, chromium, and welding fumes compared to non-exposed workers. | Moderate |
|  |  | S2. Do the collected data allow to address the research questions? | Yes. The authors used LDCT results from 6,326 workers, alongside detailed job history and smoking data, to address the distribution across exposure groups. |  |
|  |  | 3.1. Are the participants representative of the target population? | Partially. The study included large number of participants (n = 6,326). However, single shipyard sample, exclusion of women, and a relatively young population limit generalizability. |  |
|  |  | 3.2. Are measurements appropriate regarding both the outcome and intervention (or exposure)? | Yes. Standardized LDCT protocol for screening and Lung-RADS for nodule classification. Exposure was based on job category. |  |
|  |  | 3.3. Are there complete outcome data? | Yes. Primary outcome data are sufficiently complete. A minor loss to follow-up (11.4% among Lung-RADS ≥3) was explicitly noted. |  |
|  |  | 3.4. Are the confounders accounted for in the design and analysis? | Yes. Key confounders (age and smoking pack-years) were adjusted for. |  |
|  |  | 3.5. During the study period, is the intervention administered (or exposure occurred) as intended? | Yes. Exposure was determined by job type and longest-held role |  |
| Zhang et al. (38) | Retrospective cohort study | S1. Are there clear research questions? | Yes. The study clearly aims to evaluate LDCT-detected lung cancer characteristics among hospital employees.. | Moderate |
|  |  | S2. Do the collected data allow to address the research questions? | Yes. Large multi-centre dataset (LDCT n=8,392) with relevant clinical variables. |  |
|  |  | 4.1. Is the sampling strategy relevant to address the research question? | Yes. The study used a large multicenter convenience sample of 8,392 hospital employees who volunteered to undergo LDCT screening, which is relevant for the study. |  |
|  |  | 4.2. Is the sample representative of the target population? | Partially. While representative of hospital employees in those regions, the sample is skewed toward females (70.7%) and nonsmokers (91.4%), which may limit generalizability to the general population. |  |
|  |  | 4.3. Are the measurements appropriate? | Yes. Standardized screening modality (LDCT) and detection rate of pathologically confirmed lung cancer as outcome measure. |  |
|  |  | 4.4. Is the risk of nonresponse bias low? | Partially. Participation rate was 53.5%. However, the basic characteristics of the source population are reported, partially mitigating concerns. |  |
|  |  | 4.5. Is the statistical analysis appropriate to answer the research question? | Yes. Analyses appropriately describe detection rates and clinical characteristics.  Pearson chi-squared test was utilized to compare the lung cancer detection rate. |  |
| Billatos et al. (39) | Prospective cohort study | S1. Are there clear research questions? | Yes. The study aims to develop/validate biomarkers to discriminate indeterminate nodules (DECAMP-1) and predict future lung cancer (DECAMP-2). | Moderate |
|  |  | S2. Do the collected data allow to address the research questions? | Yes. Large prospective cohorts (DECAMP-1 and DECAMP-2) with clinical data, imaging, and various biospecimens (nasal, bronchial, blood, urine) collected to address diagnostic and predictive objectives. |  |
|  |  | 3.1. Are the participants representative of the target population? | Partially. The cohort is predominantly composed of male military personnel, among whom the prevalence and burden of smoking are substantially higher than in the general population. |  |
|  |  | 3.2. Are measurements appropriate regarding both the outcome and intervention (or exposure)? | Yes. Use of validated methods: LDCT-detected nodules, adjudicated cancer diagnosis, and molecular biomarkers from multiple biospecimens |  |
|  |  | 3.3. Are there complete outcome data? | Not applicable. This is a study protocol; outcomes (e.g., cancer diagnosis, biomarker performance) are not yet available. However, potential risk of attrition and loss to follow-up is acknowledged. |  |
|  |  | 3.4. Are the confounders accounted for in the design and analysis? | Can’t tell. Prospective design with standardized data collection, but no explicit analytical plan for confounder adjustment detailed |  |
|  |  | 3.5. During the study period, is the intervention administered (or exposure occurred) as intended? | Yes. Standardized multicentre protocol with defined inclusion criteria, biospecimen collection, and adjudication process ensures consistency. |  |
| Welch et al. (40) | Prospective cohort Study | S1. Are there clear research questions? | Yes. The study clearly aims to determine if incorporating occupational exposure into screening algorithms results in a lung cancer prevalence similar to smoking-only criteria. | Moderate |
|  |  | S2. Do the collected data allow to address the research questions? | Yes. Data includes LDCT results, smoking history, and detailed occupational exposure data from 1290 participants. |  |
|  |  | 3.1. Are the participants representative of the target population? | Yes. Participants consisted of a large sample drawn from the BTMed program, specifically targeting workers at high risk due to construction site exposures. |  |
|  |  | 3.2. Are measurements appropriate regarding both the outcome and intervention (or exposure)? | Yes. The study used standardized LDCT protocols for screening and ACR-LungRADS for nodule classification. Radiation doses were appropriately monitored via Dose Length Product (DLP) and SF-12 questionnaire for health status. |  |
|  |  | 3.3. Are there complete outcome data? | Yes. The study reports outcomes for all 1,290 baseline scans, including follow-up data, and details the stage/type of all cancers detected. |  |
|  |  | 3.4. Are the confounders accounted for in the design and analysis? | No. There was no formal statistical adjustment for possible confounders (e.g., age, smoking intensity, etc.). |  |
|  |  | 3.5. During the study period, is the intervention administered (or exposure occurred) as intended? | Yes. Screening protocol (LDCT) applied consistently, with eligibility criteria based on NCCN recommendation |  |
| Markowitz et al. (41) | Prospective cohort study | S1. Are there clear research questions? | Yes. The objective is clearly stated: to determine lung cancer screening yield and stages in a union-sponsored program for high-risk nuclear weapons workers. | Good |
|  |  | S2. Do the collected data allow to address the research questions? | Yes. The study collected comprehensive data on CT scan results, lung cancer staging, smoking history, and occupational risk factors for 7,189 workers. |  |
|  |  | 3.1. Are the participants representative of the target population? | Partially yes. Large occupational cohort across multiple sites, but non-random selection limit generalizability |  |
|  |  | 3.2. Are measurements appropriate regarding both the outcome and intervention (or exposure)? | Yes. The study used standardized LDCT protocols. Cancer staging followed I-ELCAP protocols and was read by a senior thoracic radiologist. Validated occupational and smoking history assessments. |  |
|  |  | 3.3. Are there complete outcome data? | Yes. The study reports on all 80 detected lung cancers, including cell type and stage. Annual scan compliance was 83%. |  |
|  |  | 3.4. Are the confounders accounted for in the design and analysis? | Yes. Regression analyses were conducted to adjust for key confounders. |  |
|  |  | 3.5. During the study period, is the intervention administered (or exposure occurred) as intended? | Yes. Screening program implemented consistently across sites using consistent LDCT protocol. |  |
| Barbone et al. (42) | Prospective cohort study | S1. Are there clear research questions? | Yes. The study explicitly evaluates whether LDCT screening could reduce lung cancer (LC) mortality in a high-risk asbestos-exposed worker population. | Good |
|  |  | S2. Do the collected data allow to address the research questions? | Yes. The authors used mortality data (all-cause and cause-specific) and cancer incidence linked through regional health databases and cancer registries over a 10-year period. |  |
|  |  | 3.1. Are the participants representative of the target population? | Yes. The study participants, drawn from a formal occupational surveillance cohort of asbestos-exposed workers, included a fairly large sample (n= 2,433) that is representative of the target population. |  |
|  |  | 3.2. Are measurements appropriate regarding both the outcome and intervention (or exposure)? | Yes. Intervention was LDCT screening. Outcomes (mortality and cancer incidence) were retrieved via validated registry record linkage. |  |
|  |  | 3.3. Are there complete outcome data? | Yes. The study utilized record linkage with regional databases for vital status and cancer occurrence, and individuals lost to follow-up were excluded from the final study population |  |
|  |  | 3.4. Are the confounders accounted for in the design and analysis? | Yes. The authors used Cox proportional hazard models adjusted for age, smoking history, asbestos exposure level and comorbidities |  |
|  |  | 3.5. During the study period, is the intervention administered (or exposure occurred) as intended? | Yes. Screening was systematic for the LDCT-P group; 98.6% of participants had a second LDCT within two years of the baseline. |  |
| Abtahi et al. (43) | Cross-sectional study | S1. Are there clear research questions? | Yes. The objective is clearly stated: to determine if serum CEA and CYFRA 21-1 levels in workers exposed to respirable synthetic vitreous fiber (SVF) and dust can indicate progression toward lung cancer. | Good |
|  |  | S2. Do the collected data allow to address the research questions? | Yes. The researchers measured serum levels of the markers and compared them between exposed workers and a control group. |  |
|  |  | 3.1. Are the participants representative of the target population? | Yes. The study included nearly all eligible workers (145 out of 150) from a specific glass wool company, providing a representative sample of that specific high-risk occupational group. |  |
|  |  | 3.2. Are measurements appropriate regarding both the outcome and intervention (or exposure)? | Yes. Exposure was measured using standardized air sampling (NIOSH Method 7400a). Outcomes (serum markers) were measured using validated ELISA kits. |  |
|  |  | 3.3. Are there complete outcome data? | Yes. Biomarker data obtained for all included participants |  |
|  |  | 3.4. Are the confounders accounted for in the design and analysis? | Partially. Individuals with medical histories that could skew results (e.g., liver disease, asthma, other malignancies) were excluded and sub-analyses to account for smoking were performed. However, exposure to other workplace carcinogens (e.g., formaldehyde) was not controlled. |  |
|  |  | 3.5. During the study period, is the intervention administered (or exposure occurred) as intended? | Yes. This was an observational study of natural occupational exposure. Exposure likely heterogeneous and not controlled. |  |
| Kato et al. (44) | Cross-sectional study | S1. Are there clear research questions? | Yes. The study aims to evaluate the efficacy of LDCT screening specifically for subjects with asbestos exposure. | Good |
|  |  | S2. Do the collected data allow to address the research questions? | Yes. Data on CT findings (cancer, plaques, asbestosis) were collected and categorized by the level of asbestos exposure. |  |
|  |  | 3.1. Are the participants representative of the target population? | Yes. A large multicenter sample (n = 2132) of relevant high-risk occupational group. However, predominantly male (96.2%) and older population (mean 76 years). |  |
|  |  | 3.2. Are measurements appropriate regarding both the outcome and intervention (or exposure)? | Yes. Exposure (asbestos) was assessed via occupational history , and outcomes (lung cancer, mesothelioma) were measured using standardized LDCT protocols. |  |
|  |  | 3.3. Are there complete outcome data? | Yes. The study clearly reports the number of subjects screened and the detected cases. |  |
|  |  | 3.4. Are the confounders accounted for in the design and analysis? | Yes. Adjusted for smoking history, fibrotic + emphysema changes and pleural effusion. |  |
|  |  | 3.5. During the study period, is the intervention administered (or exposure occurred) as intended? | Yes. All participants underwent the same LDCT screening protocol as intended by the study design |  |
| Smargiassi et al. (45) | Cross-sectional study | S1. Are there clear research questions? | Yes. The study explicitly aims to evaluate the validity of chest ultrasonography as an additional diagnostic tool for managing asbestos-related diseases. | Moderate |
|  |  | S2. Do the collected data allow to address the research questions? | Yes. The researchers collected comparative data between chest ultrasonography and the "gold standard" LDCT to calculate sensitivity and specificity. |  |
|  |  | 3.1. Are the participants representative of the target population? | Partially. The cohort consisted of randomly selected retired asbestos-exposed workers under surveillance. However, the inclusion of only 59 out of 12,622 eligible persons limits generalizability. |  |
|  |  | 3.2. Are measurements appropriate regarding both the outcome and intervention (or exposure)? | Yes. Exposure (asbestos) was documented via occupational history; Outcomes were measured using standardized LDCT and US protocols. |  |
|  |  | 3.3. Are there complete outcome data? | Yes. Findings for all 59 participants were reported for both the index and reference tests with no reported dropouts. |  |
|  |  | 3.4. Are the confounders accounted for in the design and analysis? | No. While age and exposure duration were recorded, these key confounders were not adjusted for. |  |
|  |  | 3.5. During the study period, is the intervention administered (or exposure occurred) as intended? | Yes. Ultrasonography appears systematically performed following a standardized, blinded protocol. |  |
| Gaballah et al. (46) | Cross-sectional study | S1. Are there clear research questions? | Yes. The study aims to assess wood dust genotoxicity and evaluate sputum PCR as a screening tool for early lung cancer prediction. | Good |
|  |  | S2. Do the collected data allow to address the research questions? | Yes. Researchers gathered environmental wood dust levels, clinical manifestations, genotoxicity markers (CA, SCE, Comet assay), and gene methylation data. |  |
|  |  | 3.1. Are the participants representative of the target population? | Partially yes. Although the participants are representative of the high-risk occupational group, all exposed workers were from one factory only, thus limiting external validity. |  |
|  |  | 3.2. Are measurements appropriate regarding both the outcome and intervention (or exposure)? | Yes. Environmental exposure measured quantitatively (8-hour Time Weighted Average (TWA)) via personal and stationary monitors. Outcomes measured via valid lab methods and standardized protocols (comet assay, CA, SCE, PCR). However, no direct clinical lung cancer outcomes. |  |
|  |  | 3.3. Are there complete outcome data? | Yes. Data were reported for all 86 subjects (43 exposed, 43 control) without mention of attrition or missing laboratory results. |  |
|  |  | 3.4. Are the confounders accounted for in the design and analysis? | Yes. Strict exclusion criteria addressed chemical co-exposures, chronic diseases (diabetes/hypertension), family cancer history, and heavy smoking. The control group was matched for age and sex. |  |
|  |  | 3.5. During the study period, is the intervention administered (or exposure occurred) as intended? | Yes. Wood dust exposure was confirmed to exceed national and international permissible limits. |  |
| Okereke et al. (47) | Retrospective review | S1. Are there clear research questions? | Yes. The study clearly aims to report the results of a lung cancer screening program at a Veterans Affairs medical center (VAMC) and compare them with historic (pre-implementation) results to understand the program's effects. | Moderate |
|  |  | S2. Do the collected data allow to address the research questions? | Yes. The authors collected demographics, CT scan results, pathologic data (stage/histology), and survival data for both the screening and prescreening periods to measure changes in detection rates and cancer staging.. |  |
|  |  | 3.1. Are the participants representative of the target population? | Yes. Study included a large sample of veterans meeting high-risk criteria (age 55–74, heavy smoking history), which representative of the target population. |  |
|  |  | 3.2. Are measurements appropriate regarding both the outcome and intervention (or exposure)? | Yes. Outcomes (lung cancer detection rate and stage) were measured via standard, validated clinical methods: LDCT screening. |  |
|  |  | 3.3. Are there complete outcome data? | Yes. The study accounts for all 1,832 patients screened and provides a clear breakdown of nodule detection, biopsy results, and histologies for those diagnosed with cancer. |  |
|  |  | 3.4. Are the confounders accounted for in the design and analysis? | No. No adjustment for confounders, only comparison with historical cohort. |  |
|  |  | 3.5. During the study period, is the intervention administered (or exposure occurred) as intended? | Yes. Screening implementation clearly defined (eligibility: age 55–74, ≥30 pack-years, LDCT annually). The program used a standardized protocol involving EMR clinical reminders, nurse coordinators, and multidisciplinary boards to ensure eligible veterans were identified and managed consistently. |  |
| Felten et al. (48) | Prospective cohort study | S1. Are there clear research questions? | Yes. The study clearly aimed to determine the practicability and efficacy of combining LDCT and sputum cytology for early lung cancer detection in asbestos-exposed workers. | Moderate |
|  |  | S2. Do the collected data allow to address the research questions? | Yes. The researchers collected clinical data from screenings (LDCT and sputum) and followed up with participants to confirm diagnoses, allowing for the calculation of sensitivity, specificity, and predictive values. |  |
|  |  | 3.1. Are the participants representative of the target population? | No. High risk participants of asbestos-exposed power industry workers, representing only 4.2% (n=187) of those examined (n = 4446) and 3.3% of the total cohort (n =5632). |  |
|  |  | 3.2. Are measurements appropriate regarding both the outcome and intervention (or exposure)? | Yes. standardized methods: LDCT and cytology. Outcomes were confirmed through clinical work-up and a three-year follow-up period |  |
|  |  | 3.3. Are there complete outcome data? | Yes. Clear report on all 187 participants who fulfilled the admission criteria. They explicitly accounted for exclusions (n=76) due to refusal or lack of usable sputum samples, ensuring the final analysis was based on complete data sets for the target subgroup. |  |
|  |  | 3.4. Are the confounders accounted for in the design and analysis? | Partially. Major confounders for lung cancer—age, tobacco consumption, and duration of asbestos exposure—were used directly in the risk stratification formula to identify the study population. This ensured the analysis was focused on a group where these factors were pre-accounted for in the selection process. However, no adjustment in the analysis. |  |
|  |  | 3.5. During the study period, is the intervention administered (or exposure occurred) as intended? | Yes. The surveillance was conducted according to a structured protocol. Participants received detailed instructions for sputum collection, and LDSCT scans were read by experienced radiologists and re-read by occupational physicians to ensure consistency. |  |
| Mastrangelo et al. (49) | Program evaluation | S1. Are there clear research questions? | Yes. The study aims to evaluate the outcomes of a long-term (2000–2011) health surveillance program for asbestos workers and propose a refined diagnostic protocol. | Moderate |
|  |  | S2. Do the collected data allow to address the research questions? | Yes. The authors collected clinical data, including radiology (X-ray/CT) and pathological diagnoses, which directly inform the effectiveness of the surveillance. |  |
|  |  | 4.1. Is the sampling strategy relevant to address the research question? | Yes. High-risk workers identified through institutional lists and local health authorities, covering the intended population of formerly exposed workers |  |
|  |  | 4.2. Is the sample representative of the target population? | Partially. The first phase has a participation rate of 58% (1,165/2,000). Further surveillance program was on-demand. |  |
|  |  | 4.3. Are the measurements appropriate? | Yes. Used standardized ILO classifications for chest X-rays and Low-Dose CT (LDCT). Exposure was quantified using validated questionnaires with specific job modules. |  |
|  |  | 4.4. Is the risk of nonresponse bias low? | No. Voluntary participation with no analysis differences between responders and non-responders, or reasons for non-participation. |  |
|  |  | 4.5. Is the statistical analysis appropriate to answer the research question? | Yes. Descriptive statistics (frequencies and percentages) were used to track diagnostic trends over an 11-year period, which is appropriate for a descriptive surveillance report. |  |
